# Supplementary material for: Age, Loneliness, and Social Media Use in Adults during COVID-19: A Latent Profile Analysis
Source: Int J Environ Res Public Health. 2023 May 27;20(11):5969. doi: 10.3390/ijerph20115969 (PMC10252391; doi:10.3390/ijerph20115969)
Supplement: Supplementary file 1 [file ijerph-20-05969-s001.zip › ijerph-2311555-supplementary.pdf]

Age, Loneliness, and Social Media Use in Adults during COVID-19: A Latent Profile Analysis

Supplemental Materials

Principal Component Analysis of the Technology Barriers Questionnaire

Construct validity of the Technology Barriers Questionnaire was evaluated using a principal component analysis (PCA) with orthogonal rotation (varimax). Results identified four components (i.e., eigenvalues over Kaiser's criterion of 1) among the 25 items, which in combination explained 69.86% of the variance. The Kaiser-Meyer-Olkin measure verified the sampling adequacy for this analysis, KMO = 0.95, and Bartlett's test of sphericity χ^2 (300) = 18182.70, $p < 0.001$, indicated the correlations between items were sufficient for PCA. Table S1 shows the component values after rotation. Component values were used to group items and form component scores. Three items showed high scores for more than one component and were not included in any of the component scores. Analysis of the items suggests component 1 (5 items) reflects barriers associated with time and money, component 2 (10 items) represents barriers due to knowledge, component 3 (3 items) barriers due to sensory and motor limitations, and component 4 (4 items) reflects concerns over privacy.

Table S1. *Results of the Principal Component Analysis of the Technology Barriers Questionnaire*

	**1**	**2**	**3**	**4**
	**Time/Money**	**Knowledge**	**Sensory/Motor**	**Privacy**
I do not use technology because it slows me down	**0.75**	0.19	0.31	0.15
I do not use technology because it is too fast, and I cannot keep up	**0.75**	0.35	0.26	0.14
I do not use technology because it creates more time-consuming problems than I would otherwise experience	**0.79**	0.29	0.20	0.22
I do not use technology because it is too expensive	**0.81**	0.20	0.15	0.19
I do not use technology because I cannot afford it	**0.75**	0.17	0.22	0.16
I often find the technology too complex to use	0.41	**0.69**	0.14	0.23
I find others know more about technology than I do	0.15	**0.76**	-0.03	0.25
I do not know enough about technology to use it effectively	0.49	**0.64**	0.17	0.18

The constant developments and upgrades in the technology are a burden for me	0.43	**0.60**	0.09	0.35
I am typically behind younger persons in my family in technology I use	0.09	**0.78**	0.06	0.17
If young people are residents in 'technology land', I may be considered an immigrant	0.10	**0.76**	0.14	0.21
I do not believe that I will ever be able to learn how to properly use technology	0.39	**0.66**	0.31	0.15
I do not use technology because I have a hard time following user manuals or instructions	0.48	**0.58**	0.40	0.08
I do not use technology as much as I would like because I do not have people in my life who can help me with my technological questions	0.39	**0.57**	0.41	0.16
When I ask others to help me with technology, they do it for me instead of helping me learn how to do it myself	0.18	**0.56**	0.46	0.22
I do not use technology as much as I would like because I have problems with my vision or hearing	0.31	0.26	**0.75**	0.06
I do not use technology as much as I would like because I have problems moving my hands	0.29	0.18	**0.84**	0.07
I do not use technology as much as I would like because I have problems with memory and/or thinking	0.31	0.19	**0.79**	0.06
It bothers me that things I do with technology could be traced even years from now	0.18	0.26	0.10	**0.82**
I feel that my use of technology makes it easier to invade my privacy	0.10	0.30	0.06	**0.82**
I feel technology is too intrusive on my personal life	0.54	0.19	0.12	**0.64**
I do not use technology because others could use it to monitor me	0.52	0.23	0.26	**0.51**
I am better at understanding and using technology than younger people*‡	0.09	0.47	-0.58	-0.21
Using technology blurs boundaries between home life and other activities‡	0.55	0.09	0.15	0.50
I do not want to use technology because I much prefer human interaction‡	0.49	0.27	0.04	0.50

Bold indicates the highest component score or the score used to classify the items int
*This item was reverse-scored before the total score was computed. ‡Items were not included in component scores.

Bivariate Correlations among the Primary Study Variables

Table S2. *Correlation Coefficients for Relations among the Primary Study Variables.*

	Social Media Use	Technology Barriers	UCLA Loneliness
Age	-0.22**	0.10**	0.29**
Social Media Use	-	-0.22**	-0.01
Technology Barriers			0.31**

**. Correlation is significant at the 0.01 level (2-tailed).

Post-hoc between group analyses for the Technology Barriers Questionnaire Total Score.

Table S3. Post-hoc Kruskal-Wallis Test Comparing Profiles on the Total Technology Barriers Score

**Sample 1 vs. Sample 2**	**Test Statistic**	**Std. Error**	**Std. Test Statistic**	**Sig.**	**Adj. Sig.**
Profile 2 vs. Profile 5	-151.418	25.703	-5.891	0.000	0.000
Profile 2 vs. Profile 3	-239.158	28.961	-8.258	0.000	0.000
Profile 2 vs. Profile 1	263.301	27.251	9.662	0.000	0.000
Profile 2 vs. Profile 5	-293.230	33.570	-8.735	0.000	0.000
Profile 5 vs. Profile 3	87.740	26.451	3.317	0.001	0.009
Profile 5 vs. Profile 1	111.883	24.567	4.554	0.000	0.000
Profile 5 vs. Profile 4	141.812	31.430	4.512	0.000	0.000
Profile 3 vs. Profile 1	24.143	27.958	0.864	0.388	1.000
Profile 3 vs. Profile 4	-54.072	34.146	-1.584	0.113	1.000
Profile 1 vs. Profile 4	-29.929	32.708	-0.915	0.360	1.000

*Each row tests the null hypothesis that the Sample 1 and Sample 2 distributions are the same. Asymptomatic significances (2-sided tests) are displayed. The significance level is 0.050. Significance values have been adjusted by the Bonferroni correction for multiple tests.
